# Short-Term Detraining Alters Body Composition and Lipid Profile but Not Performance in Recreational University Swimmers

**DOI:** 10.3390/sports14060246

**Published:** 2026-06-17

**Authors:** Foteini Dantsi, Antigoni Kypraiou, Nikolaos Kouvelas, Vasiliki Manou, Dimitrios C. Milosis, Dimitrios Loupos, Anatoli Petridou

**Affiliations:** School of Physical Education and Sport Science, Aristotle University of Thessaloniki, 54124 Thessaloniki, Greece; fdantsis@auth.gr (F.D.); ankypraiou@auth.gr (A.K.); nikoskou@auth.gr (N.K.); vmanou@phed.auth.gr (V.M.); dmylosis@phed.auth.gr (D.C.M.); loupo@phed.auth.gr (D.L.)

**Keywords:** biochemical assessment, fat mass, lean mass, physiological adaptations, students, swimming, training

## Abstract

Most detraining research in swimming has focused on competitive athletes, whereas less is known about recreational university swimmers, a population commonly exposed to temporary interruptions in structured training. This study examined the effects of 4 weeks of naturally occurring detraining on anthropometric, body composition, biochemical, kinematic, and performance variables in recreational university swimmers. Sixteen young swimmers were assessed before and after detraining, following at least one year of participation in a structured university swimming training program. Anthropometric, body composition, biochemical, kinematic, and performance variables were assessed before and after the detraining period. After detraining, waist and hip circumferences, fat mass, and total cholesterol increased. In contrast, fasting glucose, triglycerides, post-exercise lactate, 50 m performance, and kinematic variables showed no statistically significant changes. These findings suggest that, in recreational university swimmers, anthropometric, body composition, and metabolic variables may be more sensitive to short-term detraining than sprint performance-related outcomes. However, the absence of statistically significant performance changes should be interpreted with caution.

## 1. Introduction

Regular participation in structured exercise induces favorable anthropometric, metabolic, and performance-related adaptations in young adults [1,2]. In university populations, exercise interventions have been associated with improved body composition, cardiometabolic profile, cardiorespiratory fitness, and physical performance [3,4,5]. Among various exercise modalities, swimming is a widely practiced activity with documented benefits for cardiorespiratory fitness, body composition, blood lipids, and broader health outcomes in adults and non-elite swimmers [6,7,8]. Despite these well-documented benefits of systematic exercise, university students exhibit considerable variability in physical activity levels and physical fitness [9]. Academic demands, examination periods, and holiday breaks may disrupt regular participation in structured exercise, leading to inconsistent training adherence and temporary interruptions in training [10,11]. Such interruptions may result in the partial or complete loss of previously acquired adaptations, a process known as detraining [12].

The magnitude and time course of detraining depend on the duration of training cessation, the physiological outcome examined, and the individual’s previous training status. Long-term detraining (≥8 weeks) has been associated with reductions in maximal oxygen uptake and muscle oxidative capacity, along with adverse alterations in metabolic function, body composition, lipid profile, and performance [13,14,15]. Short-term detraining (≤4 weeks) appears to induce more subtle, yet still measurable, changes, particularly in cardiorespiratory and metabolic adaptations [14,16,17]. Evidence from endurance-based and mixed endurance-power sports, such as soccer and kayaking, further indicates that short periods of reduced or absent training can increase body fat and impair physical fitness, fat-free mass, strength, or aerobic power [18,19]. Although these sports differ from swimming in their technical and biomechanical demands, they support the broader concept that aerobic, metabolic, and body composition adaptations may be sensitive to interruptions in regular training.

In swimming, most detraining evidence derives from competitive athletes. Approximately 4–5 weeks of detraining have been associated with increases in body mass, waist circumference, or body fat percentage, as well as reductions in VO_2_peak and resting metabolic rate in competitive swimmers [20,21,22]. Short-term detraining has also been linked to altered blood lactate responses during standardized swimming trials, suggesting changes in metabolic efficiency [23,24,25]. Some studies in adolescent or young highly trained swimmers have reported reductions in stroke rate (SR) and swimming performance after short-term detraining [20,21,25], whereas others have found no changes in stroke rate, stroke length, stroke index, or swimming velocity in younger swimmers [26]. These inconsistencies may be related to differences in age, training status, previous training volume, detraining duration, and the distance or intensity of the performance test.

Taken together, previous research on short-term detraining has focused mainly on highly trained or competitive athletes, whereas evidence in recreational university swimmers remains scarce. This distinction is important because recreational university swimmers typically train at lower volumes, may have different technical proficiency and physiological reserves, and are more likely to experience lifestyle changes during holidays and examination periods.

The primary aim of the present study was to investigate the multidimensional effects of 4 weeks of training cessation during the Christmas holidays and subsequent examination period on anthropometric characteristics, body composition, biochemical variables, kinematic variables, and swimming performance in recreational university swimmers. A secondary aim was to examine the associations of biochemical, kinematic, and performance variables with anthropometric and body composition variables. We hypothesized that short-term detraining would lead to unfavorable changes in body composition and metabolic markers and could be accompanied by changes in swimming performance and stroke efficiency indices. We further hypothesized that biochemical, kinematic, and performance variables would be associated with anthropometric and body composition variables.

## 2. Materials and Methods

### 2.1. Participants

The study sample consisted of 16 recreational university swimmers (7 males and 9 females), aged 21.4 ± 1.5 years, with a mean height of 1.73 ± 0.11 m (mean ± standard deviation, SD). According to the participant classification framework of McKay et al. [27], the participants were classified as Tier 1, corresponding to recreationally active individuals, thereby distinguishing them from trained, highly trained, or elite competitive swimmers. The sample size was calculated a priori using the G∗Power software (version 3.1.9.2, Kiel University, Kiel, Germany) [28]. The analysis indicated that a sample size of 12 participants was required to detect significant effects with a large within-participant effect size (dz = 0.9), α = 0.05, and power = 0.80. This effect size was selected based on a previous detraining study in swimmers reporting large within-participant effects for performance-related outcomes [20]. To account for possible dropouts, we included 16 participants.

The inclusion criteria were as follows: (i) healthy university students, (ii) regular participation in a structured university fitness swimming program for at least one year, (iii) no participation in any competitive sport for at least five years prior to the study, (iv) no participation in any other type of structured exercise, and (v) willingness to maintain their habitual diet throughout the study period. The exclusion criteria included (i) any musculoskeletal injury that prevented participation in the swimming training program and (ii) any diagnosed medical condition or use of medication that could affect the measured biochemical variables. Eligibility criteria, including the absence of participation in competitive sport or any other type of structured exercise, were verified by self-report during participant screening. Participants were also instructed to refrain from any structured exercise during the 4-week detraining period.

The research was conducted in accordance with the ethical principles of research on human participants, as outlined in the Declaration of Helsinki. The participants were informed about the purpose and procedures of the study and provided written consent. Approval of the research was granted by the Ethics Committee of the School of Physical Education and Sport Science at Thessaloniki, Aristotle University of Thessaloniki (approval number 104/12-1-2022).

### 2.2. Study Design

**Figure 1:**
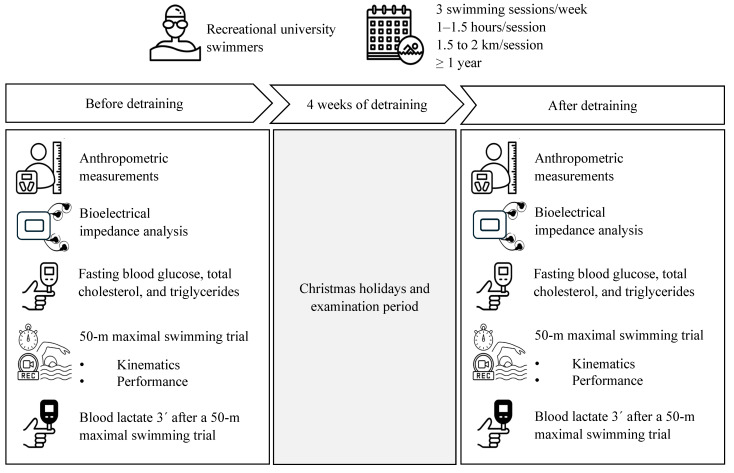
Study design. See text for details.

This study employed a prospective, longitudinal, repeated-measures design. The participants were assessed twice. The first assessment was conducted after at least one year of participation in a university swimming fitness training program, immediately prior to the Christmas holidays. Briefly, the swimming training program consisted of three 1–1.5 h sessions per week, with each session covering 1.5 to 2 km, resulting in a total weekly training volume of 4 to 6 km. The second assessment was performed following a 4-week detraining period. At each assessment, anthropometric measurements and body composition analysis were performed, and fasting blood glucose, total cholesterol, and triglycerides were measured. Participants then performed a 50 m maximal freestyle swimming trial in an indoor 25 m pool, during which swimming performance and kinematic variables were assessed. Blood lactate concentration was measured 3 min post-exercise. The participants were instructed to maintain their habitual diet throughout the study and to refrain from structured exercise during the 4-week detraining period. However, free-living physical activity and dietary intake were not objectively monitored. All measurements were performed under standardized conditions at both time points, using the same procedures, equipment, pre-assessment instructions, testing order, time of day, pool environment, and investigators. The study design is shown in Figure 1.

### 2.3. Anthropometric Measurements

Body mass and height were measured using an electronic scale with an integrated stadiometer (Seca, Hamburg, Germany) to the nearest 0.1 kg and 0.01 m, respectively, with minimal clothing and no shoes on. Body mass index (BMI) was then calculated from body mass and height. Furthermore, waist and hip circumferences were measured using a non-extendable tape to the nearest 0.5 cm, and the waist-to-hip ratio was calculated from these measures. All anthropometric measurements were performed by the same trained assessor at both time points.

### 2.4. Assessment of Body Composition

Body composition was assessed through single-frequency bioelectrical impedance analysis with a Bodystat 1500 device (Isle of Man, UK). Participants were instructed to abstain from alcohol, caffeine, and energy drink intake for 24 h, from vigorous exercise for 12 h, and from food or fluid intake for at least 4 h prior to testing. Measurements were carried out at room temperature, with participants in the supine position, legs slightly abducted, and arms positioned away from the trunk. Electrodes were placed on the right hand (at the wrist and above the middle finger) and right foot (at the ankle and above the second toe), as recommended by the manufacturer, and a current at a frequency of 50 kHz was applied. The following variables were obtained: fat mass (kg, %), lean mass (kg, %), and total body water (TBW) (L, %).

### 2.5. Kinematic Analysis and Performance Assessment

Kinematic and performance assessments were conducted in an indoor 25 m swimming pool. The participants performed a 300 m low-intensity swimming warm-up, followed by a 50 m maximal freestyle swimming trial, initiated with a push-off from the wall. All testing sessions took place between 9:00 and 12:00. Ambient air temperature and relative humidity were 28.5 °C and 65%, respectively.

For kinematic analysis, the swimming trial was recorded using a Sony 8 mm Hi8 video camera (Sony, Tokyo, Japan) operating at a sampling frequency of 25 Hz and mounted on a fixed tripod. Video recordings were analyzed using the Windows Movie Maker software (version 16.4.3528, Redmond, WA, USA). Stroke rate, SL, and SI were determined from the time required to complete three consecutive stroke cycles at the midpoint (12.5 m) of each 25 m swimming segment, according to the equations described by Bielec and Makar [29].

For the performance assessment, the split time for each 25 m segment and the total 50 m time were recorded using an electronic stopwatch. Swimming velocity was subsequently calculated. Immediately after the swimming trial, participants were asked to rate their perceived exertion (RPE) using the Borg 6–20 scale [30]. All video analyses and timing procedures were performed by the same trained assessor/investigator at both time points.

### 2.6. Assessment of Biochemical Variables

Glucose, total cholesterol, triglycerides, and post-exercise lactate concentrations were measured in capillary blood using an automatic portable analyzer (Accutrend^®^ Plus, Roche Diagnostics, Rotkreuz, Switzerland). The Accutrend Plus system has previously been evaluated for the assessment of glucose, total cholesterol, and triglycerides in adults, and portable Accutrend lactate analyzers have shown acceptable reliability for blood lactate measurement, supporting their use in field-based assessments [31]. Specific test strips were used for each biochemical variable according to the manufacturer’s instructions. Capillary blood samples were obtained via transcutaneous puncture of the fingertip using disposable lancets. The first drop of blood was discarded, and subsequent drops were used for the analyses. Glucose, total cholesterol, and triglycerides were measured prior to the swimming trial in the fasted state, while lactate was measured 3 min after the 50 m maximal swim.

### 2.7. Statistical Analysis

Data are presented as the mean ± SD. The normality of data distribution was assessed using the Shapiro–Wilk test. Depending on the distribution, data were analyzed using a paired-samples Student’s *t*-test or the Wilcoxon signed-rank test. For the main pre–post comparisons, 95% confidence intervals (CIs) for the within-participant differences were also calculated and reported where applicable. Effect sizes (ES) for the Student’s t-test were determined as Cohen’s dz and were classified as small (0.20), medium (0.50), or large (0.80), according to Cohen [32]. Effect sizes for the Wilcoxon signed-rank test were determined as r and were classified as small (0.10), medium (0.30), or large (0.50) [32].

Associations between variables measured at both time points were examined using general linear models, with participant ID and time point included as fixed factors to account for the non-independence of repeated observations within participants. In these models, biochemical, kinematic, or performance variables were entered as dependent variables, while anthropometric and body composition variables were entered as predictors. The level of statistical significance was set at α = 0.05. All statistical analyses were performed using SPSS version 29.0 (IBM, Armonk, NY, USA).

## 3. Results

### 3.1. Anthropometric and Body Composition Variables

Table 1 presents the anthropometric characteristics of the participants before and after 4 weeks of detraining. Following detraining, waist and hip circumferences increased significantly, with medium-to-large effect sizes, whereas body mass, BMI, and waist-to-hip ratio did not change significantly.

**Figure 2:**
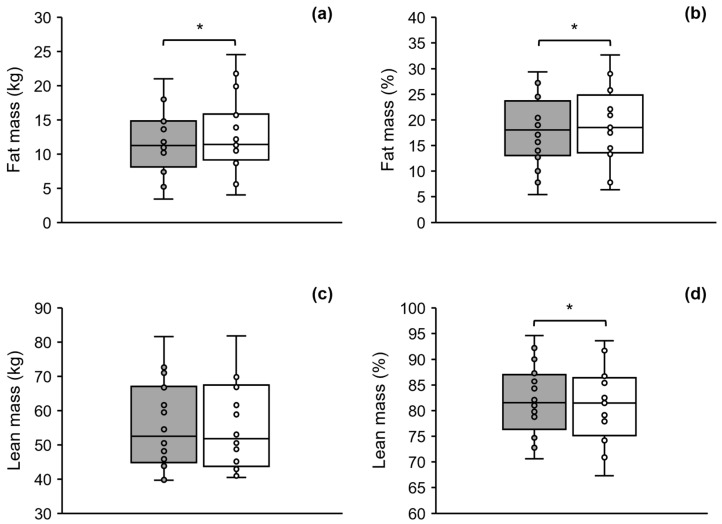
Box plots of (**a**,**b**) fat mass and (**c**,**d**) lean mass before (gray boxes) and after (open boxes) a 4-week detraining period in recreational university swimmers. Each box represents the interquartile range, and the center line represents the median. Whiskers are extended to the most extreme data point that is no more than 1.5 times the interquartile range from the edge of the box (Tukey style). Dots represent individual values. * Significant differences between time points (*p* < 0.05).

Regarding body composition, fat mass increased significantly after 4 weeks of detraining, both in absolute terms (*p* = 0.023, Cohen’s dz = 0.634; Figure 2a) and relative to body mass (*p* = 0.012, Cohen’s dz = 0.715; Figure 2b). In contrast, relative lean mass decreased significantly (*p* = 0.012, Cohen’s dz = 0.715; Figure 2d), while absolute lean mass did not change significantly (*p* = 0.218, Cohen’s dz = 0.321; Figure 2c). Relative TBW also decreased significantly from 57.2 ± 5.5% to 56.2 ± 6.1% (*p* = 0.009, Cohen’s dz = 0.756), whereas absolute TBW did not change significantly (38.7 ± 8.3 vs. 38.2 ± 8.1 L; *p* = 0.060, Cohen’s dz = 0.509).

### 3.2. Kinematic and Performance Variables

Table 2 presents the kinematic and performance variables during the 50 m maximal freestyle swimming trial before and after 4 weeks of detraining. No significant changes were found in any kinematic or performance variable. Similarly, RPE remained similar at approximately 14 on the Borg 6–20 scale at both time points (*p* = 0.566, Cohen’s dz = 0.147).

### 3.3. Biochemical Variables

Fasting total cholesterol concentration increased significantly from before to after detraining, with a large ES (157.8 ± 11.4 vs. 166.5 ± 19.6 mg/dL; *p* = 0.047, r = 0.497). In contrast, no significant changes were found in fasting glucose (71.9 ± 15.3 vs. 70.9 ± 11.2 mg/dL; *p* = 0.826, Cohen’s dz = 0.056), fasting triglycerides (84.0 ± 23.7 vs. 90.1 ± 42.9 mg/dL; *p* = 0.824, r = 0.056), or post-exercise lactate concentrations (8.24 ± 3.49 vs. 8.36 ± 3.59 mmol/L; *p* = 0.858, Cohen’s dz = 0.045).

### 3.4. Association Analyses

After accounting for repeated measurements within participants, no physiologically meaningful associations were found between biochemical, kinematic, or performance variables and anthropometric or body composition variables.

## 4. Discussion

To date, detraining research has predominantly focused on elite athletes, with limited evidence available for non-competitive populations. The present study aimed to address this gap by examining the effects of short-term detraining in recreational university swimmers, a population frequently exposed to interruptions in structured training, thereby providing ecologically valid evidence in a real-world setting. Specifically, we investigated the effects of a 4-week training cessation period on anthropometric characteristics, body composition, biochemical markers, kinematic variables, and performance. Our main findings indicate that (i) short-term detraining led to increases in waist and hip circumferences, accompanied by an increase in fat mass; (ii) total cholesterol concentration increased following detraining; and (iii) kinematic and performance variables during a 50 m maximal freestyle swimming trial remained unchanged.

### 4.1. Anthropometric and Body Composition Responses

Our results showed a 1.2 cm increase in waist circumference (1.7%) following 4 weeks of detraining. Waist circumference is widely recognized as a health-related anthropometric marker, and abdominal obesity has been associated with all-cause mortality [33]. In overweight or obese adults, each 1 cm increase in waist circumference has been associated with an approximately 2% higher relative risk of a cardiovascular disease event [34]. Furthermore, a large prospective study reported a 7% and 9% higher mortality risk for each 5 cm increment in waist circumference in adult overweight men and women, respectively [33]. However, these findings should be used only as contextual evidence for the relevance of waist circumference as a cardiometabolic risk marker. The clinical relevance of the small short-term increase observed in the present study should be interpreted cautiously, given the small sample size, the healthy young population, and the short duration of detraining. Rather than indicating an immediate increase in cardiovascular risk, this finding suggests that even a brief interruption in structured swimming training may be accompanied by measurable unfavorable changes in central adiposity-related markers. Our finding of increased waist circumference following short-term detraining is consistent with those reported in a study of young competitive swimmers following a similar period of training cessation [22]. The absence of change in waist-to-hip ratio can be explained by the concomitant increase in hip circumference, which was of similar magnitude to the increase in waist circumference.

Regarding body weight, the change found in response to 4 weeks of training cessation reflects body recomposition, as fat mass increased by 0.8 kg, whereas lean body mass showed a non-significant decrease of 0.3 kg. Similarly, Ormsbee et al. [22] reported an increase in fat mass with no change in lean body mass following 5 weeks of detraining in young competitive swimmers. However, in contrast to our findings, they found a significant increase in body mass, probably due to a nearly two-fold higher increase in fat mass compared to our study (1.8 vs. 0.8 kg, respectively). This discrepancy could be attributed to differences in training volume. Specifically, the participants in this study trained 3 to 4.5 h per week before detraining, whereas those in the study by Ormsbee et al. trained more than 10 h per week. Cessation of higher-volume training, without a corresponding reduction in energy intake, may create a greater positive energy balance and lead to larger increases in fat mass and body mass. The absence of significant changes in lean body mass in the present study suggests that such changes may become more apparent after longer periods of detraining.

### 4.2. Biochemical Responses

The observed increase in fasting total cholesterol concentration of approximately 9 mg/dL, corresponding to a 5.7% increase after the 4-week detraining period, suggests that training cessation was accompanied by an unfavorable change in lipid profile. However, this finding should be interpreted cautiously, given the borderline statistical significance, small sample size, and possible influence of diet and daily routines. Elevated total cholesterol, particularly ≥240 mg/dL, has been associated with increased risk for cardiovascular disease in young adults aged between 20 and 38 years [35]. Total cholesterol concentration remained within reference ranges following detraining in the present study. Therefore, the observed increase should not be overinterpreted, although it may become clinically relevant if sustained over time. The increase in total cholesterol following training cessation is physiologically plausible, as regular aerobic and combined exercise reduces total cholesterol, according to a recent systematic review and meta-analysis [36]. Thus, removal of the training stimulus may partially reverse these lipid adaptations. Our findings are in line with a study investigating the effect of long-term detraining (2 months) in collegiate elite taekwondo athletes, which reported an 8.3% increase in total cholesterol [37]. However, this comparison should be interpreted cautiously because the detraining period in that study was longer than in the present study, and the participants differed in sport type and training status. In contrast, a study of collegiate competitive swimmers reported no change in total cholesterol following 5 weeks of detraining [22]. These contrasting findings may be related to differences in population characteristics, training status, previous training volume, detraining duration, and lifestyle factors during the detraining period.

The absence of significant changes in fasting triglyceride concentrations in the present study is consistent with findings reported in collegiate competitive swimmers after 5 weeks of detraining [22]. In contrast, an increase in triglyceride concentration has been reported in amateur cyclists following a longer detraining period (2 months) [15]. These contrasting findings suggest that alterations in triglycerides may require extended periods of training cessation or a substantial reduction in predominantly endurance-based exercise to become apparent. Similarly, we found no change in fasting blood glucose concentration following short-term detraining. This finding likely reflects the robustness of glucose regulatory mechanisms, which appear resilient to brief periods of training cessation in healthy young adults.

Our findings of no difference in lactate after the 50 m maximal swimming trial following 4 weeks of detraining are in contrast with studies of competitive swimmers following similar periods of training cessation [20,23,24,25]. Specifically, increases in blood lactate concentration have been reported after 3 × 20 m, 200 m, and 400 m swimming trials following 4–5 weeks of detraining in competitive swimmers [20,23,24,25]. One plausible explanation for this discrepancy may be differences in the training status of the participants. Highly trained athletes may be more vulnerable to the rapid loss of adaptations related to lactate production or clearance during periods of training cessation. However, this explanation was not directly tested in the present study and should therefore be interpreted cautiously.

### 4.3. Kinematic and Performance Responses

In the present sample, 4 weeks of training cessation were not associated with detectable impairments in kinematic variables or 50 m sprint swimming performance. However, this finding should be interpreted with caution, as the relatively small sample size increases the possibility of type II error. Moreover, some performance-related variables showed small-to-moderate effect sizes in the direction of slightly reduced swimming performance or efficiency. Therefore, although these changes were not statistically significant, they may still be of practical relevance, particularly in more performance-oriented populations.

Our findings are partly consistent with those of a study in children aged approximately 10 years with at least 12 months of competitive swimming training, which reported no changes in SR, SL, SI, or 200 m performance after detraining [26]. In contrast, most studies in young, highly trained, or competitive swimmers have reported reductions in SR and swimming performance after similar periods of detraining, with or without changes in SL and SI [20,21,25]. These contrasting findings may be due to differences in training status, previous training volume, technical proficiency, age, and the distance or intensity of the performance test. Thus, rather than indicating that swimming performance is unaffected by detraining, the present findings suggest that sprint swimming performance may be relatively more resistant to short-term detraining in recreational swimmers. Importantly, performance outcomes do not always directly reflect underlying physiological or body composition changes, particularly in short-duration sprint tasks. Swimming performance is multifactorial and may be influenced by technical skill, neuromuscular coordination, motivation, and familiarization with the test. In addition, the 50 m maximal swimming trial may have limited sensitivity for detecting short-term physiological decline in recreational swimmers. Performance in this task relies heavily on technique and coordination, which may be relatively resilient over a short detraining period. Therefore, subtle adverse changes in body composition or metabolic profile may occur before they are translated into detectable impairments in sprint performance.

### 4.4. Limitations

A limitation of the present study is that free-living physical activity, sedentary behavior, and habitual diet were not objectively assessed, although participants were instructed to refrain from structured exercise and maintain their usual diet throughout the study period. This is particularly relevant because the detraining period coincided with the Christmas holidays and the examination period, during which energy intake, eating patterns, daily physical activity, and sedentary behavior may change. However, the study was designed to examine a naturally occurring interruption of structured swimming training under real-world conditions. Therefore, dietary records or intensive lifestyle monitoring were not implemented, as these procedures could have increased participant awareness and potentially altered their usual behavior during this period. This approach preserved the ecological validity of the study but limited our ability to isolate the independent contribution of detraining from concurrent changes in diet and daily routines. Consequently, the increases in fat mass and total cholesterol cannot be attributed exclusively to training cessation, as concurrent changes in diet, physical activity, or other lifestyle factors may have contributed. Additionally, the lipid profile assessment was limited to total cholesterol and triglycerides, precluding a more comprehensive analysis of lipoprotein subfractions and limiting the interpretation of the increase in total cholesterol.

Another limitation is that body composition was assessed using single-frequency BIA. Although pre-assessment conditions were standardized to reduce the potential influence of hydration status, BIA estimates remain dependent on body water distribution and hydration state. Therefore, this method may have limited sensitivity for detecting small changes in body composition over time. More precise methods, such as dual-energy X-ray absorptiometry, could provide more accurate estimates of fat mass and lean mass changes in future detraining studies.

Regarding performance and kinematic analysis, the participants were recreational university swimmers with relatively low baseline performance compared with competitive swimmers, which should be considered when extrapolating the findings to higher-performance athletic populations. Additionally, the use of 25 Hz video recording, non-specialized software, and manual stopwatch timing may have limited the detection of small changes in sprint performance and stroke-related variables. Although procedures were standardized across time points, future studies should use higher-frequency video, specialized motion analysis software, and automated timing to improve measurement accuracy. Finally, the absence of a non-exercising control group did not allow us to exclude the possibility that the observed changes in outcome measures were due to factors other than detraining.

The association analyses were conducted in a relatively small sample, and therefore, the absence of physiologically meaningful associations between the variables examined should be interpreted cautiously. The study may have been underpowered to detect small-to-moderate but potentially meaningful changes, particularly in kinematic and performance-related variables, because the a priori sample size calculation was based on a large expected effect size. Consequently, the non-significant findings for swimming performance and stroke mechanics should be interpreted as an absence of statistically detectable changes in the present sample, rather than as definitive evidence that short-term detraining has no effect on these outcomes. Future studies with larger samples are needed to confirm these findings and to better estimate the magnitude of smaller detraining effects in recreational swimmers.

### 4.5. Practical Implications

For university fitness programs, the present findings highlight the importance of maintaining some level of structured activity during holiday or examination periods. Even short interruptions in structured swimming programs may be accompanied by unfavorable changes in body composition and total cholesterol, even when sprint swimming performance appears unchanged. Coaches and university fitness program coordinators may therefore consider implementing simple maintenance strategies during academic breaks, such as brief structured swimming or general exercise sessions and encouragement of regular physical activity. In addition, because short sprint performance tests may not immediately reflect subtle changes in body composition or metabolic profile, simple anthropometric and body composition assessments may provide useful complementary information when monitoring recreational swimmers.

## 5. Conclusions

Four weeks of detraining were accompanied by unfavorable changes in selected anthropometric, body composition, and metabolic variables in recreational university swimmers, including increases in waist and hip circumferences, fat mass, and total cholesterol. In contrast, no significant changes were detected in kinematic variables, swimming performance, or lactate response to maximal swimming exercise. These findings suggest that, in the present sample, anthropometric, body composition, and metabolic variables appeared more sensitive to short-term detraining, whereas sprint performance-related outcomes may be relatively more resistant. However, the observed changes were small and should be interpreted cautiously, particularly given the short duration of detraining, the relatively small sample size, and the possibility that small but practically meaningful performance changes may not have been detected.

## Figures and Tables

**Table 1:** Anthropometric characteristics before and after 4 weeks of detraining (n = 16, mean ± SD).

	Before	After	Mean Difference (95% CI)	Two-Sided *p* Value	ES
Body mass (kg)	67.7 ± 13.6	68.2 ± 13.2	−0.5 (−1.3 to 0.4)	0.268	0.288
Body mass index (kg/m^2^)	22.3 ± 2.7	22.5 ± 2.7	−0.2 (−0.5 to 0.1)	0.223	0.318
Waist circumference (cm)	77.5 ± 10.6	78.7 ± 10.7	−1.2 (−1.7 to −0.6)	**<0.001**	1.034
Hip circumference (cm)	96.0 ± 7.5	97.9 ± 7.1	−1.9 (−3.2 to −0.5)	**0.009**	0.749
Waist-to-hip ratio	0.80 ± 0.09	0.80 ± 0.08	0.00 (−0.01 to −0.02)	0.357	0.238

Mean difference refers to the pre-detraining minus post-detraining value. CI, confidence interval; ES, effect size (as Cohen’s dz) following Student’s *t*-test. Boldface indicates significant outcomes (*p* < 0.05).

**Table 2:** Kinematic and performance variables before and after 4 weeks of detraining (n = 16, mean ± SD).

	Before	After	Mean Difference (95% CI)	Two-Sided *p* Value	ES
SR in the first 25 m (cycles/m)	37.5 ± 4.2	37.0 ± 5.7	0.5 (−1.1 to 2.0)	0.559	0.149
SR in the second 25 m (cycles/m)	35.2 ± 4.3	35.5 ± 5.3	−0.3 (−1.7 to 1.1)	0.622	0.126
Velocity in the first 25 m (m/s)	1.17 ± 0.17	1.14 ± 0.16	0.03 (−0.02 to 0.08)	0.167	0.363
Velocity in the second 25 m (m/s)	1.03 ± 0.15	1.02 ± 0.18	0.01 (−0.04 to 0.05)	0.743	0.084
Velocity in 50 m (m/s)	1.09 ± 0.16	1.07 ± 0.16	0.02 (−0.02 to 0.05)	0.283	0.279
SL in the first 25 m (m)	1.88 ± 0.24	1.86 ± 0.24	0.02 (−0.06 to 0.10)	0.587	0.139
SL in the second 25 m (m)	1.76 ± 0.23	1.73 ± 0.25	0.03 (−0.06 to 0.12)	0.537	0.158
SI in the first 25 m (m × m/s)	2.23 ± 0.53	2.13 ± 0.45	0.10 (−0.05 to 0.24)	0.174	0.357
SI in the second 25 m (m × m/s)	1.83 ± 0.41	1.79 ± 0.48	0.04 (−0.12 to 0.19)	0.617	0.128
Time in the first 25 m (s)	21.7 ± 3.0	22.3 ± 3.0	−0.6 (−1.5 to 0.4)	0.202	0.334
Time in the second 25 m (s)	24.9 ± 4.0	25.2 ± 4.4	−0.3 (−1.6 to 0.9)	0.563	0.148
Time in 50 m (s)	46.9 ± 6.9	47.6 ± 7.1	−0.7 (−2.3 to 1.1)	0.435	0.201

Mean difference refers to the pre-detraining minus post-detraining value. CI, confidence interval; ES, effect size (as Cohen’s dz) following Student’s *t*-test. SR, stroke rate; SL, stroke length; SI, stroke index.

## Data Availability

The data that support the findings of this study are openly available in the repository of the Hellenic Academic Research Data Management Initiative at DOI: https://doi.org/10.26255/heal.gb82-qws3.

## Abbreviations

The following abbreviations are used in this manuscript:

BMI	Body mass index
ES	Effect size
RPE	Rate of perceived exertion
SD	Standard deviation
SI	Stroke index
SL	Stroke length
SR	Stroke rate
TBW	Total body water
